# Contextual Illusions Reveal the Limit of Unconscious Visual Processing

**DOI:** 10.1177/0956797611399293

**Published:** 2011-03

**Authors:** Julia J. Harris, D. Samuel Schwarzkopf, Chen Song, Bahador Bahrami, Geraint Rees

**Affiliations:** 1 Institute of Cognitive Neuroscience; 2 Department of Neuroscience, Physiology, and Pharmacology; 3 Wellcome Trust Centre for Neuroimaging, University College London

**Keywords:** consciousness, visual perception, cognitive neuroscience

## Abstract

The perception of even the most elementary features of the visual environment depends strongly on their spatial context. In the study reported here, we asked at what level of abstraction such effects require conscious processing of the context. We compared two visual illusions that alter subjective judgments of brightness: the simultaneous brightness contrast illusion, in which two circles of identical physical brightness appear different because of different surround luminance, and the Kanizsa triangle illusion, which occurs when the visual system extrapolates a surface without actual physical stimulation. We used a novel interocular masking technique that allowed us to selectively render only the context invisible. Simultaneous brightness contrast persisted even when the surround was masked from awareness. In contrast, participants did not experience illusory contours when the inducing context was masked. Our findings show that invisible context is resolvable by low-level processes involved in surface-brightness perception, but not by high-level processes that assign surface borders through perceptual completion.

The perceived brightness of an object does not correspond in a simple way with its luminance, and two objects of equal luminance can appear to have different levels of brightness if they are observed in different contexts. Such simultaneous brightness contrast (SBC) is observed, for example, when a gray circle appears brighter against a dark background than against a light background (Fig. 1a). Similarly, spatially distinct elements of a visual scene can induce an illusory percept of luminance contours if the organization of those elements induces the visual system to extrapolate the presence of an occluding surface (Fig. 1b), as in the Kanizsa triangle illusion (Kanizsa, 1979).

**Fig. 1.**
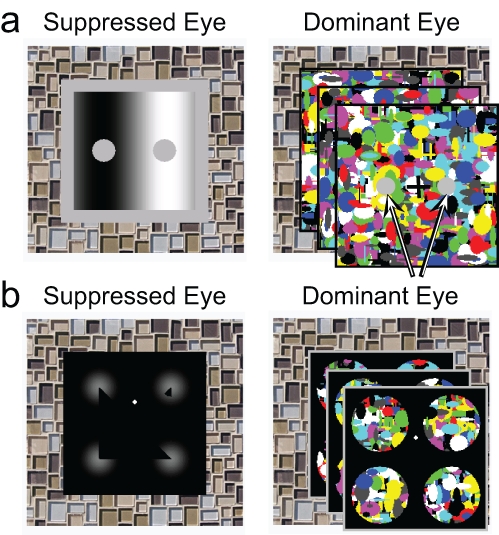
Stimuli and masks used in (a) Experiment 1 and (b) Experiment 3. For each experiment, an example stimulus is shown on the left, and a continuous flash suppression (CFS) mask is shown on the right. Experiment 1 utilized the simultaneous brightness contrast illusion. The suppressed eye was shown two gray circles that were physically identical but appeared different in luminance because of the context. The CFS mask covered most of the illusion stimulus except for two “holes” (highlighted here by arrows) that coincided with the two gray circles. Experiment 3 utilized the Kanizsa triangle illusion. The suppressed eye was shown four inducer elements, three of which created the illusory impression of a triangle. In the selective-mask condition (illustrated here), four circular CFS masks covered the inducers in the illusion stimulus; in the invisible condition, a single CFS mask covered the entire stimulus.

These contextual illusions may depend on high-level inferential processes (Helmholtz, 1867; Williams, McCoy, & Purves, 1998). Alternatively, they may be mediated by low-level mechanisms operating at the earliest stages of visual processing (Mueller, 1842; Paradiso & Nakayama, 1991). These two accounts predict different illusory perception when awareness of the context is disrupted. Specifically, if high-level inferential mechanisms are involved, illusory brightness perception must require awareness of the context. Conversely, if low-level mechanisms dominate, these illusions would persist even without contextual awareness. These predictions can help draw a distinction between context-dependent perceptual phenomena that do and do not depend on awareness of the context. Such a distinction would offer the opportunity to address a fundamental question of much wider interest: What is consciousness for?

In the experiments reported here, we used a novel dichoptic masking technique to selectively render contextual parts of the stimulus invisible. Through a mirror stereoscope, participants viewed an SBC illusion or a Kanizsa triangle (Fig. 1, left) with one eye while viewing brightly colored flashing patterns (Fig. 1, right) with the other eye. These flashing patterns almost entirely overlapped the retinotopic location of the stimulus in the other eye, sparing only the locations of the illusory percept. This composite stimulus resulted in stable and selective suppression of the background context through continuous flash suppression (CFS; Tsuchiya & Koch, 2005), but left the critical portion of the stimuli phenomenally accessible to consciousness for behavioral report.

## General Method

### Participants

Twenty-two healthy, right-handed volunteers (9 male and 13 female; age range = 21–35 years) with normal or corrected-to-normal vision participated in this study. Ten volunteers took part in the first experiment, 5 in the second experiment, and 7 in the third experiment. All experiments were undertaken with the consent of the participants and approval of the local ethics committee.

### Apparatus

Stimuli were generated using the Cogent Graphics toolbox (Laboratory of Neurobiology, University College London, London, England) for MATLAB 7.2.0 (The MathWorks, Natick, MA) and were presented in a dark room on a gamma-corrected CRT display. The display had a resolution of 640 × 480 pixels in Experiments 1 and 2 and of 800 × 600 pixels in Experiment 3, and it had a refresh rate of 100 Hz in Experiments 1 and 2 and of 60 Hz in Experiment 3. With the exception of the stimuli in the first task of Experiment 1, all stimuli were viewed through a mirror stereoscope. Head and chin rests were used in all tasks to ensure a consistent viewing distance of 66 cm. Participants made responses using a computer keyboard.

## Experiment 1: SBC Under Dichoptic Masking

In Experiment 1, participants were asked to judge which of two gray circles appeared brighter in three different tasks.

### Task 1: canceling brightness differences

In the first task, each participant quantified the magnitude of the SBC illusion.

#### Stimuli

Stimuli were presented centrally within a uniform gray border (background luminance = 65 cd/m^2^) nested within a tile-frame surround (11.77° visual angle). Each stimulus consisted of two gray circles (diameter = 1.19°), one on the left side and one on the right side of a sinusoidal luminance gradient (width = 5.95°). One side of this gradient was black, and the other was white; a smooth transition from black to white in the center had a spatial frequency of 0.2 cycles per degree. The right/left position of the black and white sides of the gradient was counterbalanced across trials. One gray circle was located on the left side of the gradient and was fixed at background luminance, and the other gray circle was on the right and was assigned a random luminance value at the start of each trial.

#### Procedure

Each participant viewed the SBC stimulus binocularly. Participants were asked to use the up and down arrow keys on the keyboard to adjust the brightness of the test circle on the right to match the brightness of the reference circle on the left. When participants perceived the brightness of the two circles to be equal, they pressed the space bar to end the trial. Each participant received two blocks of six trials. Each participants’ mean values for the adjusted luminance of the test circle when it was located on the bright side and when it was located on the dark side were subsequently used in the next two phases of Experiment 1.

### Task 2: judging brightness differences

Having established the magnitude of the SBC illusion induced by our stimuli in the first task, we proceeded to investigate whether the illusion persisted when the inducing context was rendered invisible by dichoptic masking using CFS.

#### Stimuli

The stimuli for the left and right eyes were presented in a split-screen arrangement with an 8.88° horizontal displacement from the center of the screen. Each stimulus was viewed with a separate eye through a mirror stereoscope. To guide binocular fusion, we surrounded each stimulus with the same complex tile pattern used in the first task. Although this frame was identical for the two eyes, the stimuli presented to them differed: One eye was presented with a CFS mask that allowed for controlled suppression of the SBC stimulus, which was presented to the other eye (Fig. 1a).

The CFS mask consisted of randomly positioned and scaled colored rectangles and ellipses, which were regenerated at a rate of approximately 12 Hz, within a square 6.83° wide. A fixation cross was presented in the center of the CFS stimulus, with two gray circles (holes) on either side. These circles (background luminance, diameter = 1.08°) were placed so that the flashing colors of the CFS stimulus would not mask the test circles in the SBC stimulus.

The SBC stimulus consisted of two gray test circles, which participants in different conditions perceived to have different luminance, depending on the circles’ actual luminance and the luminance of the background against which they were presented.

In the illusory-difference condition, the two test circles were both set to background luminance and were presented against a sinusoidal luminance background, as in the first task. The background was again configured so that one test circle was on the bright side, and the other was on the dark side. In the real-difference condition, the two circles were of different luminance and were presented against a gray square of background luminance. The luminance values for these test circles were derived from the first task and thus were specific to each participant.

#### Procedure

Participants began each trial by pressing the space bar. They were instructed to fixate on a centrally presented cross throughout each trial, which lasted 1,200 ms. The CFS stimulus was presented to one eye for the full duration of the trial. The other eye received the test image for the first 1,000 ms and a CFS stimulus for the last 200 ms to prevent afterimages of the suppressed image. After completing each trial, participants were asked to report whether the left or the right circle was brighter (a two-alternative, forced-choice task). They were then asked whether the suppressed stimulus background was visible or invisible; they were instructed to report that the stimulus background was visible even if they saw only a small part of it for a short time. Responses to both questions were made using the left and right arrow keys on the keyboard.

Each participant completed 12 blocks of 16 trials, for a total of 96 trials in each condition. The suppressed eye, the condition, and the left/right luminance arrangement of the background in the illusory-difference condition and the circles in the real-difference condition were selected randomly for each trial but ultimately counterbalanced over each block.

### Task 3: discriminating the background

To confirm that participants’ reports that the background was invisible were really indicative of an absence of accessible information, we asked participants in a separate session to make perceptual judgments about the suppressed portion of the SBC stimulus (i.e., the background). Instead of judging which circle was brighter, as in the previous task, participants judged which side of the SBC stimulus was darker. To reduce possible cues, we configured all SBC stimuli for this task in such a way that the illusion was perceptually canceled for each participant. That is, two circles of different luminance, derived for each individual from the first task, were presented against the sinusoidal luminance gradient, with the circle on the dark side being darker than the circle on the bright side. In all other respects, the procedure was the same as in the second task.

### Results

In Task 2, the background was classed as invisible on an average of 85% of trials in both the real-difference and the illusory-difference conditions. Only these trials were included in the analysis to preclude the possibility that trials in which part of the background was seen could have contaminated the results (the proportion of trials in which the background was visible was very low for most participants, and this precluded a reliable estimate of performance on such trials).

**Fig. 2.**
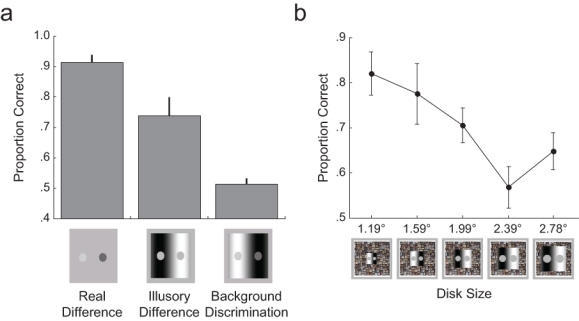
Mean proportion of correct responses in (a) Experiment 1 and (b) Experiment 2. In Task 2 of Experiment 1, participants judged which disk appeared brighter. In the real-difference condition, the two disks differed in physical brightness. In the illusory-difference condition, the two disks were the same physical brightness, and responses were judged correct if participants reported that the disk on the dark side of the stimulus was brighter than the disk on the light side. In Task 3, participants judged which side of the background was darker. The icons below the graph depict the stimuli presented to the suppressed eye. The graph in (b) shows the proportion of trials in Experiment 2 in which participants experienced simultaneous brightness contrast (i.e., they perceived the disk on the dark background as brighter than the disk on the light background) as a function of disk size. The icons below the graph depict the stimuli presented to the suppressed eye. The holes through which the gray circles appeared in the masking stimulus (not pictured) remained a constant size (smaller than the smallest disk). Error bars indicate 1 *SEM*.

In the real-difference condition, participants’ ability to distinguish which circle appeared brighter through the CFS holes was significantly better than chance (*M* = .91 correct responses), *t*(9) = 16.45, *p* < .001 (Fig. 2a). In the illusory-difference condition, perceptions that the disk on the dark side of the stimulus was brighter than the disk on the light side were also above chance frequency (*M* = .74), *t*(9) = 3.85, *p* = .004 (Fig. 2a). Thus, an invisible variation in background luminance created illusory brightness differences in a visible stimulus that were comparable with physical brightness differences.

It is important to note that in Task 3, participants could not make accurate perceptual judgments about the background (*M* = .51 correct responses), *t*(9) = 0.76, *p* = .466, and this confirms that participants were not aware of the invisible context (for this task, the background was classed as invisible in 86% of trials). Thus, brightness judgments, although critically dependent on spatial context, do not rely on awareness of that context.

The results of Experiment 1 suggested that brightness perception in SBC must arise before conscious awareness. SBC exhibits no interocular transfer (Alpern, 1963), and this fact constrains the locus of SBC to monocular neuronal populations. This observation is consistent with low-level theories of brightness perception that posit a reliance on contrast mechanisms at the border between a figure and a background, and this contrast is propagated throughout the figural region by a filling-in mechanism (Paradiso & Nakayama, 1991). We tested these theories in Experiment 2 using our dichoptic CFS masking procedure.

## Experiment 2: Border Manipulation

### Method

Starting with the same experimental configuration as in the illusory-difference condition of Experiment 1, we varied the size of the circles (1.19°, 1.59°, 1.99°, 2.39°, and 2.78°) in the SBC stimuli but kept the size of the holes constant (1.08°) in the CFS stimulus. To keep the ratio of contextual background to test circle constant, we also varied the size of the background (3.58°, 4.76°, 5.95°, 7.13°, and 8.30°, respectively) in the SBC stimuli, but we kept the size of the CFS stimulus mask and the tile frame constant (8.59° and 11.77°, respectively; Fig. 2b). The CFS mask was presented to one eye, and the SBC stimulus was presented to the other, as in Experiment 1. Participants were asked to judge which circle was brighter in two blocks of 16 trials for each circle size.

### Results

There was no significant difference in the percentage of trials on which the background was classed as invisible as a function of circle size—1.19°: 88% invisible; 1.59°: 78% invisible; 1.99°: = 80% invisible; 2.39°: 74% invisible; and 2.78°: 74% invisible, *F*(4, 20) = 1.46, *p* = .25. As in Experiment 1, we included only these trials in further analysis. We found that illusory perception decreased significantly, *F*(4, 20) = 4.435, *p* = .01, with increasing circle size (Fig. 2b), which indicates that mechanisms at the border are indeed important for this illusion.

## Experiment 3: Kanizsa Triangle Under Dichoptic Masking

At what level of abstraction is consciousness necessary for visual processing of spatial context? In Experiment 3, we used our dichoptic masking procedure to test whether a presumably high-level process, the perception of illusory contours through amodal completion (a Kanizsa triangle), could also occur without contextual awareness. We examined whether perception of illusory contours persisted when the inducing stimulus elements were masked from awareness through CFS.

### Method

#### Stimuli

Kanizsa triangle stimuli were generated by placing four inducer elements in a square configuration (width = 9.16°) on a black background (Fig. 1b). In order to facilitate masking of these elements by CFS, we rendered them as a Gaussian luminance profile (*SD* = 0.92°) to avoid sharp edges. A black wedge was removed from each of these inducer elements. Two of these wedges measured 45°; a third wedge measured 90°. The wedges extended to the edge of their respective inducers, and were positioned such that they appeared to form the apexes of a right triangle. The wedge that measured 90° could be either in the bottom left or the bottom right inducer. Finally, a fourth element, opposite the hypotenuse of the illusory triangle, was a distractor inducer that was not part of the triangle. In this element, the wedge measured 45° but did not extend to the edge of the inducer. Thus, the combination of elements determined whether the Kanizsa triangle was perceived to be facing to the left or to the right.

A white fixation dot was presented 1.37° above the center of the square. We located the fixation point here in order to facilitate the perception of illusory contours, which has been shown to be stronger in the lower than in the upper visual field (Rubin, Nakayama, & Shapley, 1996). Finally, we also generated a stimulus that included only the Gaussian luminance profiles without the black wedges. All of these stimuli were presented within a tile frame similar to the one used in the other experiments but subtending 15.58° of visual angle.

As in the other experiments, the CFS mask itself was a Mondrian-like pattern of colored, randomly positioned and scaled geometric shapes that was regenerated at 10 Hz. In the selective-mask condition, we masked the four inducers but left visible the region that would be traversed by the sides of the illusory triangle (and would potentially be accessible to conscious awareness): The CFS mask was viewed only through circular apertures (diameter = 3.67°) that overlapped the inducers. In the invisible-stimulus condition, the CFS mask was 9.16° wide and thus exactly covered the area of the Kanizsa stimulus. In the visible condition, the stimulus appeared unmasked. As in the other experiments, the images for the left and right eye were presented in a split-screen arrangement with a 8.25° horizontal displacement from the center of the screen.

#### Procedure

At the start of every trial, participants were presented with a 200-ms blank stimulus, in which only the tile frame, black square, and fixation dot were visible. Then, one eye was presented with the Kanizsa triangle stimulus for 200 ms, followed by 100 ms of the stimulus containing only the Gaussian luminance profiles without the black wedges. We chose a short stimulus duration to ensure that the CFS mask adequately masked the illusion stimulus. However, we observed comparable results in pilot experiments with longer stimulus durations. As one eye viewed the Kanizsa triangle, the opposite eye was presented with the blank stimulus (visible condition), a CFS mask covering the entire Kanizsa stimulus (invisible condition), or the mask with four circular apertures covering the inducers only (selective-mask condition). Subsequently, the display for both eyes reverted back to the blank stimulus, and participants were asked to indicate using the left or right arrow keys whether they saw the Kanizsa triangle pointing to the left or to the right (a two-alternative, forced-choice task).

Participants performed 160 trials for each of the three conditions. There were equal numbers of trials with the triangle pointing left or right. The three conditions and two triangle orientations were shown in a pseudorandomized, counterbalanced order. Every 24 trials, there was a rest break, which participants terminated manually by pressing a key.

### Results

**Fig. 3.**
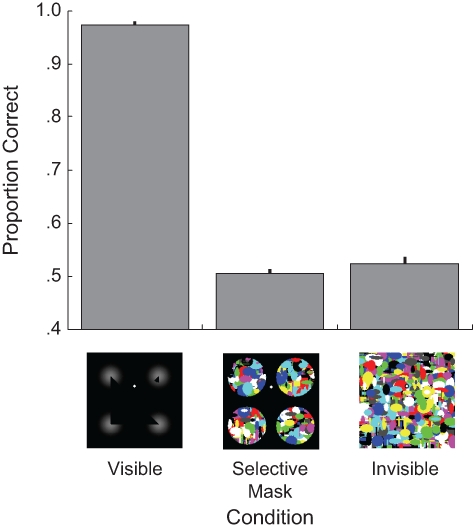
Mean proportion of correct responses in Experiment 3 as a function of condition. The three conditions, which are illustrated by the icons below the graph, all involved a Kanizsa triangle stimulus. In the visible condition, the stimulus was not masked. In the selective-mask and invisible conditions, a continuous flash suppression mask was presented in two different ways. Error bars indicate 1 *SEM*.

In the visible condition, all participants found the task easy: Performance was significantly above chance (*M* = .97 correct responses), *t*(6) = 82.44, *p* < .001. This high level of performance despite the presence of a distractor inducer shows that the brief stimulus duration was sufficient for a representation of the illusory contour to form. If the illusion also persisted without awareness of the inducers, participants’ performance should have been above chance in the selective-mask condition as well. However, as Figure 3 shows, this was not the case (*M* = .51 correct responses), *t*(6) = 0.50, *p* = .638, and performance in this condition was no different from performance when the entire stimulus was masked (invisible condition), paired *t* test, *t*(6) = –1.38, *p* = .218. Thus, the perception of illusory contours requires awareness of the inducing elements.

## Discussion

In the series of experiments reported here, we combined a novel selective-masking technique with two contextual visual illusions that modulated subjective judgments of brightness. We examined whether conscious processing of spatial context was necessary for these illusions to occur.

In Experiment 1, we found that SBC persisted even though the spatial context inducing the illusion was not consciously perceived. The high-contrast dynamic noise patterns suppressed the background region that surrounded two physically identical target circles. These target circles, which were selectively visible through the interocular mask, appeared different in brightness (Task 2) as if there were a real physical difference between the two targets (such as in Task 1). The effect was somewhat weaker for the illusory-difference than for the real-difference condition, a pattern consistent with previous findings that rendering a stimulus invisible is equivalent to reducing its luminance contrast (Blake, Tadin, Sobel, Raissian, & Chong, 2006).

In Experiment 2, we further tested whether this brightness illusion was mediated by local low-level mechanisms, in which the perceived brightness spreads from the luminance boundaries throughout the stimulus. We parametrically varied the size of the two target circles (and the surrounding backgrounds) but kept constant the size of the holes in the CFS mask through which participants viewed the illusion. This effectively changed the distance from the conscious viewing area to the luminance edges of the targets. The magnitude of the SBC depended on the size of the circles, a finding demonstrating that the illusion is indeed mediated by processes near the luminance border. Because previous work has shown that SBC is also a purely monocular process (Alpern, 1963), the neural basis of the illusion must be in the early stages of the visual pathway: the retinal ganglion cells, the lateral geniculate nucleus, or the monocular population of neurons in primary visual cortex. It is important to note that the fact that SBC occurs in monocular neurons would not necessarily rule out the possibility that it requires consciousness. Our findings therefore demonstrate that SBC is a low-level process and that it does not rely on awareness of the context.

Perception of illusory contours is also associated with the subjective experience of brightness change. It is conceivable that perception of illusory contours may involve high-level processing because the visual system should infer the presence of an occluding surface from the organization of simple visual features in the background. This may require an abstract concept of depth that may not arise without conscious analysis of the visual scene and is probably learned through experience with the environment (Walk, 1966). In Experiment 3, we tested whether visible Kanizsa inducers were necessary for the perception of illusory contours.

When not viewing stimuli through a CFS mask, participants found it easy to discriminate the shape of an illusory Kanizsa triangle; however, when the elements of the stimulus inducing the illusion were masked from awareness, behavioral performance dropped to chance levels—comparable to levels observed when the entire stimulus was masked—confirming that awareness of inducers was necessary for perception of illusory contours. Consistent with this assertion, findings of other studies have shown that patients deprived of visual experience from an early age often do not experience this illusion when their vision is restored in later life (Fine et al., 2003).

It has been suggested that illusory contours are mediated by binocular neurons (Paradiso, Shimojo, & Nakayama, 1989), a suggestion that places this effect at a higher stage of processing than the monocular mechanisms of brightness perception (Alpern, 1963; Anderson, Dakin, & Rees, 2009). Indeed, neurons responsive to illusory contour have been found in area V2 (von der Heydt, Peterhans, & Baumgartner, 1984). However, neural correlates of illusory contours induced by abutting lines have also been reported for regions as early as the primary visual cortex (Montaser-Kouhsari, Landy, Heeger, & Larsson, 2007; Ramsden, Hung, & Roe, 2001). This raises the possibility that the two types of brightness illusions—dependent on and independent from awareness of context—may not differ in their anatomical locus in the visual hierarchy. That is, they may arise in overlapping neuronal populations within V1, but illusory contours may, for example, involve predictive reentrant feedback from higher levels (Di Lollo, Enns, & Rensink, 2000; Lamme, 2003; Pascual-Leone & Walsh, 2001). In that view, the neural correlates of consciousness depend more on the process than on the anatomy involved in perception.

Taken together, our data show that perceived brightness can be influenced by invisible variations in background luminance and that such context-dependent brightness perception relies on low-level mechanisms at stimulus borders. But such influence of invisible contexts on conscious perception does not extend to all aspects of brightness perception, as the perception of illusory luminance contours requires contextual awareness. The results of these experiments support low-level theories of brightness perception and indicate that the perceptual extrapolation of global form invokes high-level inferential processes. These findings also extend the general claim— previously restricted to orientation perception (Clifford & Harris, 2005)—that unconscious context can modulate conscious perception to the realm of brightness perception. Finally, our experimental procedure of partial CFS provides a new technique with which the role of conscious perception in contextual visual processing can be investigated.
